# **Retraction: Relationship between the Autoantibody and Expression of β**_**3**_ **-Adrenoceptor in Lung and Heart**

**DOI:** 10.1371/journal.pone.0326623

**Published:** 2025-06-18

**Authors:** The PLOS One Editors

After this article [1] was published, concerns were raised regarding results presented in Figs 2, 3, and 5.

Specifically:

- In Fig 2, there appears to be partial overlap between the following panels:
  - Sham 5 Week and Non-BRL 5 Week.
  - BRL 5 Week, Sham 7 Week, and Sham 9 Week.
- There appears to be full or partial overlap between the following panels:
  - Sham 5 Week in Fig 3 in [1] and 0 week Young sham in Fig 1 in [2].
  - Non-BRL 5 Week, BRL 7 Week, and Sham 9 Week in Fig 3 in [1], and 0 week Aging heart failure and 9 Week Aging sham in Fig 1 in [2].
  - Sham 9 Week in Fig 3 in [1] and 0 week Young heart failure in Fig 1 in [2].
  - BRL 5 Week in Fig 3 in [1] and 0 Week Aging sham in Fig 1 in [2].
  - Sham 11 Week in Fig 3 in [1] and 9 Week Young sham in Fig 1 in [2].
- The following panels appear similar:
  - Fig 5A β-actin in [1] and Fig 2A β-actin in [2].
  - Fig 5C GAPDH in [1] and Fig 3A GAPDH in [2].

Regarding Figs 2 and 3, the authors stated that errors occurred in file storage. They provided replacement data for most of the panels listed above for Figs 2 and 3, however these data do not fully resolve the concerns.

The authors stated that Fig 5A β-actin in [1] and Fig 2A β-actin in [2] are the same image and that Fig 5C GAPDH in [1] and Fig 3A GAPDH in [2] are the same image, as they believed they could reuse the same GAPDH and β-actin panels as they were conducted at the same time by the same species in the same period. They stated that the Fig 5A β-actin and Fig 5C GAPDH in [1] are incorrect. They provided replacement panels for Fig 5A β-actin and Fig 5C GAPDH. The co-first author XF stated that these incorrect panels in Figs 5A and 5C did not affect the associated quantification data in Figs 5B and 5D.

In light of the extent of the above concerns that question the reliability of the results, the *PLOS One* Editors retract this article.

XF did not agree with retraction and stands by the article’s findings. GM, ZC, ML, GH, HA, ZZ, LL, JZ, and LZ either did not respond directly or could not be reached.

The Fig 3 Sham 5 Week, Non-BRL 5 Week, BRL 7 Week, Sham 9 Week, BRL 5 Week, Sham 11 Week panels, the Fig 5A β-actin panel, and the Fig 5C GAPDH panel report material previously presented in [2], published in 2010 by Taylor & Francis which are not offered under a CC BY license and are therefore excluded from this article’s [1] license.
